# The potential of high-order features of routine blood test in predicting the prognosis of non-small cell lung cancer

**DOI:** 10.1186/s12885-023-10990-4

**Published:** 2023-06-01

**Authors:** Liping Luo, Yubo Tan, Shixuan Zhao, Man Yang, Yurou Che, Kezhen Li, Jieke Liu, Huaichao Luo, Wenjun Jiang, Yongjie Li, Weidong Wang

**Affiliations:** 1 Sichuan Cancer Hospital, School of Medicine, University of Electronic Science and Technology of China, Chengdu, China; 2 School of Life Science and Technology, University of Electronic Science and Technology of China, Chengdu, China; 3 School of Medicine, Southwest Medical University, Luzhou, China; 4 Radiation Oncology Key Laboratory of Sichuan Province, Sichuan Cancer Center, Affiliated Cancer Hospital of University of Electronic Science and Technology of China, Chengdu, China

**Keywords:** Routine blood test, Lung cancer, DeepSurv model, Prognosis, High-order feature

## Abstract

**Background:**

Numerous studies have demonstrated that the high-order features (HOFs) of blood test data can be used to predict the prognosis of patients with different types of cancer. Although the majority of blood HOFs can be divided into inflammatory or nutritional markers, there are still numerous that have not been classified correctly, with the same feature being named differently. It is an urgent need to reclassify the blood HOFs and comprehensively assess their potential for cancer prognosis.

**Methods:**

Initially, a review of existing literature was conducted to identify the high-order features (HOFs) and classify them based on their calculation method. Subsequently, a cohort of patients diagnosed with non-small cell lung cancer (NSCLC) was established, and their clinical information prior to treatment was collected, including low-order features (LOFs) obtained from routine blood tests. The HOFs were then computed and their associations with clinical features were examined. Using the LOF and HOF data sets, a deep learning algorithm called DeepSurv was utilized to predict the prognostic risk values. The effectiveness of each data set’s prediction was evaluated using the decision curve analysis (DCA). Finally, a prognostic model in the form of a nomogram was developed, and its accuracy was assessed using the calibration curve.

**Results:**

From 1210 documents, over 160 blood HOFs were obtained, arranged into 110, and divided into three distinct categories: 76 proportional features, 6 composition features, and 28 scoring features. Correlation analysis did not reveal a strong association between blood features and clinical features; however, the risk value predicted by the DeepSurv LOF- and HOF-models is significantly linked to the stage. Results from DCA showed that the HOF model was superior to the LOF model in terms of prediction, and that the risk value predicted by the blood data model could be employed as a complementary factor to enhance the prognosis of patients. A nomograph was created with a C-index value of 0.74, which is capable of providing a reasonably accurate prediction of 1-year and 3-year overall survival for patients.

**Conclusions:**

This research initially explored the categorization and nomenclature of blood HOF, and proved its potential in lung cancer prognosis.

**Supplementary Information:**

The online version contains supplementary material available at 10.1186/s12885-023-10990-4.

## Introduction

Lung cancer is a global, chronic disease with a poor prognosis. The tumor–lymph node–metastasis (TNM) staging system is the most commonly used and accurate prognostic model [1], and patients may experience enhanced treatment results after obtaining the suitable treatment based on the stage. To accurately ascertain the TNM stage, patients must undergo a range of tests, such as histopathological tests, CT scans, MRI scans, and/or PET-CT scans [2]. In order to take these examinations, patients must fulfill certain criteria depending on their physical condition. Prolonged investigations in a clinical setting can often be a challenge for both patients and medical professionals, as they can last anywhere from a week to a month.

The accuracy of TNM staging in diagnosing is estimated to be around 70% [3], which is insufficient to meet the demands of clinical practice; thus, researchers are endeavoring to supplement it with easily available data. Routine blood test is distinct from other clinical examination procedures because of their ease, speed, repeatability, and capacity to track alterations over time [4, 5]. The aforementioned attributes render it a crucial factor in the diagnosis and prediction of numerous diseases, including the current COVID-19 pandemic [6]. It has been demonstrated that certain features obtained from routine blood tests, such as the Neutrophil-to-Lymphocyte Ratio (NLR), the Glasgow Prognosis Score (GPS), and the Systemic Immune-Inflammation Index (SII), can be used to predict cancer prognosis [7–9].

Researchers have discovered and continue to discover numerous complex blood features. To differentiate between the original features and the derived complex features, we can refer to them as low-order features (LOF) and high-order features (HOF) respectively. The LOF have an established naming system for their abbreviations, such as WBC (White Blood Cell Count), CRP (C-Reactive Protein), RBC_SD (RBC Distribution Width Standard Deviation), and MPV (Mean Platelets Volume). However, no such systematic system exists for HOF abbreviations, which has caused confusion in the utilization of the abbreviations of these HOFs in existing reports. For instance, the calculation formula of Systemic inflammatory marker (SIM) [10] and Systemic inflammation response index (SIRI) [7], the lung immune prognostic index (LIPI) [11] and the dNLR combined with LDH index (LNI) [12], Onodera’s prognostic nutritional index (OPNI) [13] and (Prognostic nutrition index) PNI [14] are identical, whereas the only distinction between Glasgow prognostic score (GPS) [9] and modified Glasgow prognostic score (mGPS) [14], the systemic inflammation score (SIS) and modified SIS (mSIS) [15] is their cutoff values. Moreover, the values of Lymphcyte-to-Monocyte ratio (LMR) [7] and Monocyte-to-Lymphcyte ratio (MLR) [16], Fibrinogen-to-Albumin ratio (AFR) [17] and Albumin-to-Fibrinogen ratio (FAR) [18] are inversely proportional to each other, yet their importance for prognosis remains the same when it comes to data analysis. What is more, naming with single-letter abbreviations can lead to conflicts, GLR is used as an abbreviation for Gran/Lymph [19], GGT/Lymph [20] and Glc/Lymph [21] in different documents, while LLR is an acronym for both WBC/Lymph [14] and LDH/Lymph [23].

The primary objective of this paper is to introduce the concept of high-order blood HOF and to conduct a thorough investigation of existing literature to determine its potential in predicting NSCLC prognosis.

## Methods

### Document retrieval

In order to identify as many blood HOFs as possible, a comprehensive search of articles published between January 2018 and October 2022 on PubMed was conducted. The search query was: ((“Blood Cell Count“[MeSH Terms]) OR (Complete Blood Count[Title/Abstract]) OR (“Laboratory Tests“[Title/Abstract]) OR (blood routine[Title/Abstract])) AND ((“Risk Factors“[MeSH Terms]) OR (Prognosis[MeSH Terms]) OR (Biomarkers[MeSH Terms])) AND ((complex index[Title/Abstract]) OR (ratio[Title/Abstract])) AND ((Cancer[MeSH Terms]) OR (Inflammation[Title/Abstract])) NOT ((review[Title/Abstract]) OR (Meta-analysis[Title/Abstract])).

### Patients

This research included 1,423 individuals who had been identified with lung cancer and were admitted to the Sichuan Cancer Hospital between 2015 and 2017. In line with the Chinese Medical Association’s clinical diagnosis and treatment guidelines for lung cancer [24], the treatment options for all patients were determined according to the same guidelines. This study excluded patients who had not been diagnosed with primary lung cancer or had a combination of other primary carcinomas, lacked blood test data prior to treatment, or had received anti-tumor therapy in other hospitals.

### Data collection

This study was granted approval by the Medical Ethics Committee of the Sichuan Cancer Hospital (SCCHEC-02-2021-064). Clinical and laboratory data of the patients were retrospectively obtained; histological examination was employed to verify the pathological type; and the American Joint Committee on Cancer (AJCC) Eighth Edition staging system [25] was utilized for tumor staging. The LOFs and HOFs that we used are covered in Additional Table 1. The LOFs comprise reference intervals that are considered normal.

The final follow-up, conducted in May 2021, measured overall survival (OS), which is the time from diagnosis to death caused by any cause or loss to follow-up.

### DeepSurv

To analyze both linear and nonlinear data, the DeepSurv algorithm [26], which is based on deep learning, can be employed to predict the probability of death for a particular patient. This algorithm was implemented using Python 3.7.6; for further information on the method and project code, please refer to the references [3, 26].

The input layer was set to the same dimensionality as the input data, while the three hidden layers comprised of 512, 1024, and 512 neurons respectively, and the output layer was one neuron. The experiment was trained for 500 epochs with an initial learning rate of 0.067, Adam optimizer, a decay rate of 0.06494, a discard layer loss rate of 0.2, and an L2 regularization coefficient of 0.005. The reliability of the model was evaluated using five-fold cross-validation.

To increase the DeepSurv model’s interpretability, the Shapley Additive exPlanations (SHAP) [27] approach is being utilized. The estimated importance of the features for the model was determined by using the SHAP method. For each patient, the DeepSurv model generated a predicted risk value, and a SHAP value was assigned to each feature of the patient, demonstrating the influence of each feature on the model’s output risk value.

### Statistical analysis and plotting

Statistical analysis was conducted using R version 4.0.2 (2020-06-22). Spearman’s method was applied to assess the correlation between features. The patient characteristics were generated with the help of the package “TableOne”. The “ggDCA” package was used to create decision curve analysis (DCA) curves, and “rms” nomogram was used to generate nomogram and calibration curves. Concordance index (C-index) values were used to compare the prediction and true values.

## Results

### HOFs categorize

After conducting a literature screening strategy, 1558 articles were identified, of which 1210 were suitable for analysis after filtering out those deemed unsuitable based on title and abstract screening. Through manual reading of the literature, we screened 160 HOFs and then merged them into 110 according to their calculation formula. This suggests that HOFs can be classified into three groups according to calculation method: basic proportional type (e.g. NLR and LMR), composite type (e.g. derived NLR (dNLR) and PNI), and scoring type based on the first two types (e.g. GPS and LIPI). Within this study, we identified 76 proportional (Table 1), 6 composite (Table 2), and 28 scoring (Table 3) HOFs, respectively.

**Table 1:** Proportional HOFs

	Abbreviation	Full Name	Calculation Formula	Inverse Proportional Calculation Abbreviation and Formula
1	AGR [28]; Alb/Glb [29]	Alb/Glb ratio	Alb/Glb	
2	AISI [30, 31]; PIV [32]	Aggregate index of systemic inflammation [30, 31]; The pan-immune-inflammation value [32]	Neu × Plt × Mono/Lymph	
3	AAR [33]	Alb-to-ALP ratio; Alb/ALP [34]	Alb/ALP	
4	ACrR	Albumin/Creatinine ratio [35]	Alb/Crea	
5	ALI	Advanced lung cancer inflammation index [7]	BMI×Alb×Lymph/Neu (BMI = weight[kg]/ height [m]^2^)	
6	ARR	ALP/RDW [36]	ALP/RDW	
7	ALRI [37]	AST-Lymph ratio	AST/Lymph	
8	API [33]	Age-Plt index	Age/Plt	
9	APR [34]	ALP-to-Plt ratio	ALP/Plt	
10	APRI [38, 39]	AST-to-Plt ratio index	1) (AST/(AST ULN))/Plt × 100 [38]; 2) AST/Plt [39]	
11	BLR [40]	Baso-to-Lymph ratio	Baso/Lymph	
12	CAR [41]	CRP-to-Alb ratio	CRP/Alb	Alb-to-CRP ratio [13]: Alb/CRP
13	CLR [13]; CRP/L [31]; CLS [42]	CRP-to-Lymph ratio	CRP/Lymph	LCR [29, 66]: Lymph/CRP
14	De Ritis ratio [43]; AST/ALT [44]	AST-to-ALT ratio	AST/ALT	
15	DFR [45]	D-dimer/Fib ratio	Dim/Fib	
16	d-CWL [46]		CRP/(WBC*Lymph)	
17	ELR [40]		Eosin/Lymph	
18	ENLR [47]		Eosin×Neu/Lymph	
19	FAR [18]; FARI [48]	Fib-to-Alb ratio	Fib/Alb	AFR [17]: Alb/Fib
20	FIB-4 [38]	FIB-4 index	(AST[IU/L]× Age[years])/(ALT[IU/L]^1/2^ ×Plt [10^9^/L])	
21	FPR [17, 49]		1) Fib/Plt [49] 2) Fib/preAlb [17]	
22	GLR [19–21]	1) Gran-to-Lymph ratio [19] 2) GGT to Lymph ratio [20] 3) Glc-to-Lymph ratio [21]	1) Gran/Lymph [19] 2) GGT/Lymph [20] 3) Glc/Lymph [21]	
23	GPR [50]	GGT to Plt ratio	GGT/Plt	
24	HALP [13]		HGB × Alb × Lymph/Plt	
25	HII [51]	Haematological inflammatory index [51]	Plt×Lymph/Neu×100	
26	HLAN [13]		(HGB × Lymph ×Alb/Neu)/100	
27	HLR [52]	HGB-Lymph ratio	HGB/Lymph	
28	HPR [52]	HGB-Plt ratio	HGB/Plt	
29	LA [53]		Lymph × Alb	
30	LAR [13]	Lymph-to-Alb ratio	Lymph/Alb	ALR [54]: ALP/Lymph
31	LFR [55]	LC to FIB ratio	Lymph/Fib	
32	LLR [22, 23]	1) Leukocyte Lymph ratio [22] 2) LDH to Lymph ratio [23]	1) WBC/Lymph [22] 2) LDH/Lymph [23]	
33	LMR [7]; ALC/AMC [56]	Lymph-to-Mono ratio	Lymph/Mono	MLR [17], MO/LY [57]: Mono/Lymph
34	LWR [58]	Lymphocyte-to-white blood cell ratio [58]; Absolute lymphcyte count/WBC ratios [59]	Lymph/WBC	LLR (Leukocyte Lymph ratio) [22]: WBC/Lymph
35	MAR [53]		Mono/Alb	
36	MCRP	Mono× CRP [60]	Mono× CRP	
37	MER [61]	Mono-to-Eosin ratio	Mono/Eosin	
38	MGLR [23, 62]	Monocyte/granulocyte to lymphocyte ratio	Mono× Lymph/Gran	
39	MHR [63]	Mono to HDLC ratio	Mono/HDLC	
40	MP [53]		Mono × Plt	
41	MPR [32]; MPV/PC [64]; MPV/PLT ratio [65]; MPVPCR [66]; MPVPR [47]	MPV-to-Plt ratio	MPV/Plt	PVR [67], Plt/MPV ratio [68]: PLT/MPV
42	MPctR	MPV/Pct [69]	MPV/PCT	
43	MPVLR [37, 47, 66]	MPV-to-Lymph ratio [47, 66]; MPV/lymphocytes [36]	MPV/Lymph	
44	MWR [57]	Monocyte-to-white blood cell ratio	Mono/WBC	
45	NAR [70]	neutrophil/albumin	Neu/Alb	
46	NER [71]	Neutrophil/erythrocyte ratio [71]	Neu/RBC	
47	NCRP	Neu× CRP [60]	Neu× CRP	
48	NHL [72]	The ratio of the product of Neu and HGB to lymphocytes	Neu×HGB/Lymph	
49	NHR [73]	Neu HDLC ratio	Neu/HDLC	
50	NLPR [31]; N/LP [47]	Neu to Lymph, Plt ratio	1) Neu/(Lymph × Plt) [31] 2) (Neu×100) /(Lymph×Plt) [47]	
51	NLR [40]; N/L ratio [74]; NLCR [75]; NL-R [76]; NEU/LY [77]; N:L [78]	Neu-to-Lymph ratio [40]; Neutrophil/ Lymphocyte Ratio [74]; Neutrophil-Lymphocyte count ratio [75]	Neu/Lymph	
52	NLLR	NLR/ALC [79]	Neu/Lymph^2^	
53	NM [53]		Neu × Mono	
54	NMR [70]	Neu-to-Mono ratio; Neutrophil/monocyte ratio	Neu/Mono	MNR [80]: Mono/Neu
55	NP [53]	Neu×Plt	Neu×Plt	
56	NPAR [81]	Neu percentage-to-Alb ratio	Neu_ratio/Alb	
57	NPR [13]; NPS [82]; Neu:Plt [83]; NP [53]; NLR/PLR ratio [84]	Neu-to-Plt ratio [13]; Neu-Plt score [82]; Neu:Plt score [83]; NLR/PLR [84]	Neu/Plt	PNR [85]: Plt/Neu
58	NWR [58]	Neutrophil-to-white blood cell ratio	Neu/WBC	WNR [85]: WBC/Neu
59	PAR [13]	Plt-to-Alb ratio	Plt/Alb	
60	PCRP	P-CRP [60]	Plt × CRP	
61	PDWLR	PDW/lymphocytes [36]	PDW/Lymph	
62	PDWPR	PDW/P [86]	PDW/Plt	PLT/PDW [87]: Plt/PDW
63	PPR	PDW/PCT [86]	PDW/PCT	
64	PLR [40]; PL-R [76]; TLR [88]	Plt-to-Lymph ratio [40]; Thrombocyte/Lymph ratio [88]	Plt/Lymph	
65	PMR [52]	Plt-Mono ratio	Plt/Mono	
66	PWR [89]		Plt/WBC	WPR [85]: WBC/Plt
67	RPR [90]; RDWPCR [66]	RDW-to-Plt ratio	RDW/Plt	
68	SACR	SAA/CRP ratio [91]	SAA/CRP	
69	SII [7, 72]; SIII [92]	Systemic immune-inflammation index	Neu × Plt/Lymph	
70	SIM [10]; SIRI [7, 31]; NMLR [93]	Systemic inflammatory marker [10]; Systemic inflammation response index [7, 31]; Neutrophil and monocyte to lymphocyte ratio [93]	Neu × Mono/Lymph	
71	TCHDR	TChol/HDLC [94]	TChol/HDLC×100	
72	TGHDR	TG/HDLC [94]	TG/HDLC×100	
73	ULR [95]	UA to Lymph ratio	UA/Lymph	
74	WBC/CRP [96]		WBC/CRP	
75	WHR [97]	WBC to HGB ratio	WBC/HGB	
76	WMR [98]	WBC to MPV ratio	WBC/MPV	

ULN: upper limit of normal value

**Table 2:** Composite HOFs

	Abbreviation	Full Name	Calculation Formula
1	ALBI [37, 39]	Alb-TBIL score	1) 0.66×log_10_ (TBil[µmol/L]) − 0.085×Alb[g/L] [39]; 2) 0.66×log_10_ (TBil[mg/dL]) × 17.1 − 0.085 ×Alb [g/dL] × 10 [37]
2	dNLR [12]	Derived NLR	Neu/(WBC ‒ Neu)
3	GNRI [13]	Geriatric Nutritional Risk Index	1.519 × Alb[g/L] + 41.7 × Actual body mass[kg]/Ideal body mass[kg]
4	MELD score [62]	Model for end stage liver disease	9.57×log_e_(Crea [mg/dL]) + 3.78×log_e_(TBil [mg/dL]) + 11.2×log_e_(INR)×6.43
5	PALBI [38]	Plt-Alb-TBIL (score)	{2.02 × log_10_TBil[mg/dL])} + [− 0.37×{log_10_ (TBil)}^2^] + (− 0.04 ×Alb[g/dL]) +{−3.48×log_10_ (Plt)} + [1.01 × {log_10_ (Plt)}^2^]
6	PNI [14]; OPNI [13]	Prognostic nutrition index [14]; Onodera’s prognostic nutritional index [13]	1) 10 ×Alb [g/dL] + 0.005 × Lymph[/mL][13] 2) Alb[g/L] + 5 × Lymph[10^9^/L] [14]

**Table 3:** Scoring HOFs

	Abbreviation	Name	Calculation formula	Formula details
1	GPS [41, 53]; mGPS [8, 39]; MGPS [93]	Glasgow prognostic score [41, 53]; modified Glasgow prognostic score [8, 93]	Alb score + CRP score	Assign score = 1 to each of the following: Alb < 3.5 g/dL, CRP > 10 mg/L [41, 53]; or Alb < 35 g/L, CRP > 8 mg/L [8, 39]; or Alb < 35 g/L, CRP ≥ 10 mg/L [93]
2	ACBS [8, 99]	The Aarhus composite 5 score [8, 99]	Alb score + CRP score + HGB score + Lymph score + Neu score	Assign score = 1 to each of the following: Alb ≤ 36 g/L, CRP ≥ 8 mg/L, Neu > 7 × 10^9^/L, Lymph ≤ 1.3 × 10^9^/L, and HGB < 7.3 (women) / 8.3 (men) mmol/L [99]; or Lymph < 3.5 × 10^9^/L [8]
3	CNG [8, 99]	the combination of NLR and GPS	Alb score + CRP score + NLR score	Assign score = 1 to each of the following: Alb < 35 g/L, CRP > 8 mmol/L, NLR > 2 [8]; or NLR > 1.9 [99]
4	ALB-dNLR [100, 101]		Alb score + dNLR score	Assign score = 1 to each of the following: Alb ≤ 39.6 g/L, dNLR > 1.365 [100] or Alb ≤ 40 g/L, dNLR > 1.77 [101]
5	SIS [53]; mSIS [15]	The systemic inflammation score [53]; modified SIS [15]	Alb score + LMR score	Assign score = 1 to each of the following: Alb < 39.75 g/L, LMR < 3.8 [53]; or Alb < 40 g/L, LMR < 3.4 [15]
6	A.L.A.N. score [102]		Alb score + LMR score + Neu score + NLR score	Assign score = 1 to each of the following: Alb < 35 g/L, LMR < 2.1, Neu > 7 × 10^9^/L, NLR > 3
7	INA [13]	Instant nutritional assessment	Alb score + Lymph score	Assign score = 1 for Lymph < 1.3 × 10^9^/L, score = 2 for Alb < 35 g/L
8	CONUT score [13]	the CONtrolling NUTritional status	Alb score + Lymph score + TChol score	Assign scores for Alb (0 = ≥ 3.5 g/dl; 2 = 3.0–3.49 g/dL; 4 = 2.50–2.99 g/dL; 6 = < 2.50 g/dL), Lymph (0 = ≥ 1.6 × 10^9^ G/L; 1 = 1.20–1.59 G/L; 2 = 0.80–1.19 G/L; 3 = < 0.8 G/L), TChol (0 = ≥ 180 mg/dL; 1 = 140–179 mg/dL; 2 = 100–139 mg/dL; 3 = < 100 mg/dL)
9	ANPG [103]		Alb score + Neu score	Assign score = 1 to each of the following: Alb < 35 g/L, Neu > 2 or < 7
10	Alb-NLR	Albumin-NLR [104]	Alb score + NLR score	Assign score = 1 to each of the following: Alb < 39.75 g/L, NLR ≥ 2.39
11	ALBI-PLR [105]		ALBI score + PLR score	Assign scores for ALBI (0 = <-2.60, 1 = ≥-2.6 and < -1.39, 2 =≥-1.39); for PLR (0 = ≤ 150, 1 = > 150)
12	PNI-APRI score [38]		APRI score + PNI score	Assign score = 1 to each of the following: APRI > 0.98, PNI < 46.5
13	Baso-NLR [106]		Baso score + NLR score	Assign score = 1 to each of the following: Baso < 15 × 10^6^/L, NLR > 2.585
14	Baso-PLR [106]		Baso score + PLR score	Assign score = 1 to each of the following: Baso < 15 × 10^6^/L, PLR > 232.5
15	PI [93]	Prognostic index	CRP score + WBC score	Assign score = 1 to each of the following: CRP ≥ 10 mg/L, WBC ≥ 11 × 10^9^/L
16	LIPI [7]; LNI [12]	The lung immune prognostic index [7]; dNLR combined with LDH index [12]	dNLR score + LDH score	Assign score = 1 to each of the following: dNLR > 3, LDH > ULN [7]; or dNLR > 1.985, LDH > 244U/L [12])
17	dNLR-PNI [107]	the combination of dNLR and PNI	dNLR score + PNI score	Assign score = 1 to each of the following: dNLR > 1.7, PNI < 46
18	Eosin-NLR [106]		Eosin score + NLR score	Assign score = 1 to each of the following: Eosin < 95 × 10^6^/L, NLR > 2.585
19	Eosin-PLR [106]		Eosin score + PLR score	Assign score = 1 to each of the following: Eosin < 95 × 10^6^/L, PLR > 232.5
20	F-NLR [108]	a combination of NLR and Fib	Fib score + NLR score	Assign score = 1 to each of the following: Fib > 3.4 g/dL, NLR > 4.1 [103]
21	COP-LMR [109, 110]	the combination of Plt and LMR	LMR score + Plt score	1) Assign score = 1 to each of the following: Plt > 221 × 10^9^ /L, LMR < 3.96) [109]; 2) Assign score = 1 to each of the following: Plt > 30 × 10^4^ /µL, LMR < 3.6 [110]
22	MLR-NLR [111]		MLR score + NLR score	Assign score = 1 to each of the following: MLR > 0.36, NLR > 2.77
23	coNLR-PDW [112]	the combination of NLR and PDW	NLR score + PDW score	Assign score = 1 to each of the following: NLR > 1.59, PDW > 13.0
24	cNPS [113]; NLR-PLR score [114]	the combination of NLR and PLR	NLR score + PLR score	Assign score = 1 to each of the following: NLR > 2.461, PLR > 248.4 [114]
25	COP-NLR [115, 116]	the combination of Plt and NLR	NLR score + Plt score	1) Assign score = 1 to each of the following: Plt < 15 × 10^4^ /µL, NLR ≥ 2.0) [115] 2) Assign score = 0 for (Plt level < 300 × 10^9^ /L and NLR < 3); score = 1 for (Plt level ≥ 300 × 10^9^ /L and NLR < 3); and score = 2 for (NLR ≥ 3) [116]
26	PIV-LDH [32]		PIV score + LDH score	Assign score = 1 to each of the following: PIV > 366, LDH < 1.5×ULN
27	PLR + PNI [117]		PLR score + PNI score	Assign score = 1 to each of the following: PLR ≥ 150, PNI < 45
28	Co-PaL [118]	the combination of preAlb and Lymph	PreAlb score + Lymph score	Assign score = 1 to each of the following: preAlb < 180 mg/L, Lymph < 1.5 × 10^9^/L)

To avoid similar issues in the future, we have proposed a set of rules for the naming of blood HOFs, with the aim of providing researchers with a consistent and accurate nomenclature. These rules include, but are not limited to:

1. Preference should be given to the abbreviations reported in Tables 1, 2 and 3 of this article, and it is advised to use the abbreviations with more reports in the left column, rather than the reverse proportional mode on the right.
2. Use abbreviations of terms related to clinical relevance. Although both the Lung Immune Prognostic Index (LIPI) and the dNLR combined with LDH index (LNI) are identical, it is suggested to use the LIPI due to its more accurate representation.
3. The product type feature is denoted by the initial letter of the feature, whereas the proportion type feature is indicated by the combination of the initial letter and the suffix ‘R’, indicating Ratio. For example, LA stands for the product of lymphocytes and albumin, while LAR is the ratio of lymphocytes to albumin.
4. The nomenclature of proportional features shall be based on the order of obtaining the ratio that is greater than one. It is important to note that multiplying the coefficient should be avoided when adjusting the value, as this will not alter the significance of this feature in data analysis.
5. In the event of a clash in naming with a single acronym, the second letter or full name of the conflicting feature should be employed. When the abbreviation of GLR is unclear, GlcLR can be used to signify the Glucose-to-Lymphocyte ratio and GranLR for the Granulocyte -to-Lymphocyte ratio.
6. It is advisable to limit the number of abbreviated names to between 3 and 6 characters to avoid confusion.

### Patient characteristics

Following the acquisition of HOFs, we immediately collected patient data for validation. The cohort included 1423 individuals with NSCLC, with 945 having adenocarcinoma and 478 having squamous cell carcinoma. At diagnosis, the majority of patients were in the later stages, with 482 in stage III and 595 in stage IV. Approximately 36% (51/1423) of the patients were either current or former smokers. The number of men (945) being almost double that of women (478). The median age was 62 years (IQR: 52–67), median follow-up was 499 days (IQR: 189-1162.5). Upon follow-up, 675 (47.4%) patients had died. The baseline characteristics of the study cohort are outlined in Additional Table 2.

### Correlation analysis

Having thoroughly explored the reported HOFs, we proceeded to investigate whether there is any correlation between each blood feature and other clinical characteristics, including sex, age, staging, smoking status, and pathological type, in order to gain further understanding. It should be noted that, as many patients in our cohort did not have a blood biochemical test prior to treatment, the HOFs in Tables 1, 2 and 3 cannot be included in the analysis (Additional Table 1). To carry out a correlation analysis, we evaluated patients based on the other four parameters. The screening criteria and the features of the patients who meet the criteria are outlined in Table 4. The Spearman method was utilized to conduct correlation analysis, with a confidence interval of 0.95. All groups, except for the smoking group, consisted of 60 patients, and the correlation coefficient threshold (Rs) was set at an absolute value of 0.305. The smoking group comprised 52 patients, with a Rs of 0.321. The analysis results indicate that sex is associated with MCHC and WRPI in LOFs. The calculation formulas for the two HOFs that are related to Age already include Age, rendering their significance insignificant. Smoking can lead to an increase in neutrophils and a decrease in albumin, with a greater impact on HOFs. There was no observed correlation between blood features and pathological types. It is noteworthy that no significant correlation was observed between any LOFs and stage, but after high-order transformation, eight features were found to be related to stage. The most highly correlated feature is GGLR (GGT/Lymph), with a correlation coefficient of 0.4041. All the characteristics that exhibit correlation coefficients greater than Rs are grouped together in the final row of Table 4.

**Table 4:** The screening criteria for correlation analysis and significant results display for each group

Feature Analysed		Sex	Age	Smoking	Pathotype	Stage
Screening Critiria	Sex	30 for each sex	Female	Male	Male	Female
	Age	30–65	33–78, median 59	30–65	30–65	30–65
	Smoking	Never	Never	Never23, Smoking 29	Never	Never
	Pathotype	Aden	Aden	Aden	Aden 30, Squa 30	Aden
	Stage	I	III	I	III	15 for each stage
Significant Blood Features (r_s_^*^)		MCHC(-0.4284), WRPI (-0.323)	API (0.536), FIB4 (0.543)	Neu (-0.3608), AISI (-0.3524), AAR (0.3973), NER (-0.3693), NLPR (-0.3496), NP (-0.3468), NP (-0.3468), SII (-0.3496), COP_NLR (-0.3615)	None	ALRI (0.3334), GGLR (0.4041), GPR (0.343), HII (-0.3205), LMR (-0.3513), NHL (0.3343), SIM (0.3196), PNI_APRI (0.3421)

*R_s_ Coefficient of Rank Correlation calculated by Spearman method

### DeepSurv Analysis

To evaluate the importance of LOF and HOF data on the prognosis of lung cancer patients, models were constructed with DeepSurv algorithm and the prediction accuracy was measured by C-index. The Table 5 shows that the LOF model is relatively stable, with C-index values in the train set and the test set not significantly different. On the other hand, the HOF model can achieve a C-index value of more than 0.7 on the train set, which is comparable to the effect of staging. However, maybe due to high correlation among many HOF features, it is prone to overfitting, thereby performing poorly in the test set. Given that age, sex, and smoking status are readily available data that can be conveniently gathered during routine clinical assessments, we have grouped these three variables together as ASS. The addition of ASS (Age + Sex + Smoking) features does not enhance the prediction model’s performance significantly.

**Table 5:** The C-index of LOF- and HOF-based DeepSurv models

	C-index(95%)	
Features	Train set	Test set
**LOF**	0.6262(0.5816–0.6707)	0.6055(0.5737–0.6372)
**LOF + ASS**	0.6546(0.6415–0.6676)	0.6006(0.5616–0.6395)
**HOF**	0.7277(0.7053–0.7501)	0.5699(0.5203–0.6195)
**HOF + ASS**	0.7139(0.6972–0.7306)	0.5865(0.5455–0.6276)

**Fig. 1:**
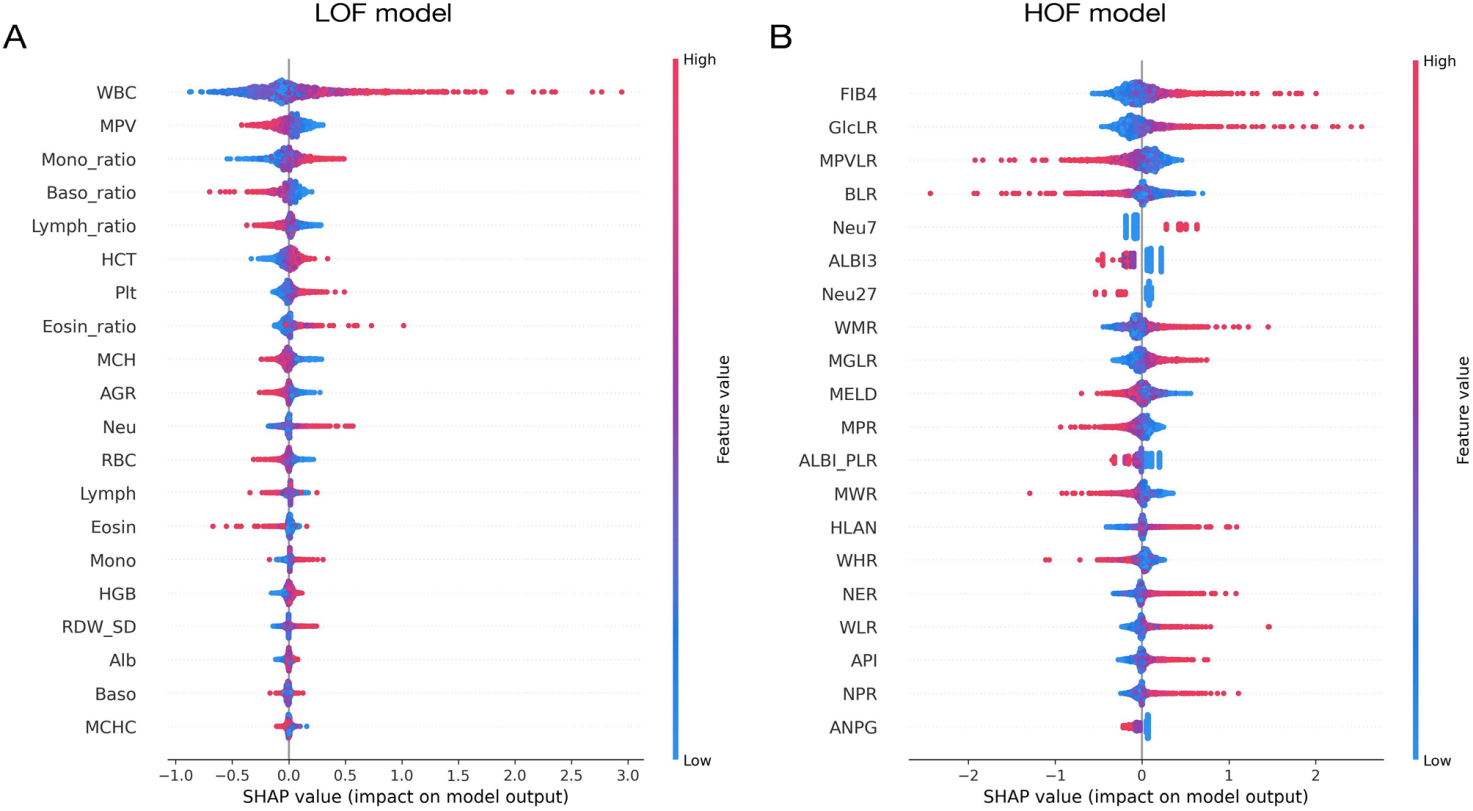
The top 20 important features in LOF- and HOF-model chosen by SHAP algorithm. A: LOF model. B: HOF model

To evaluate the impact of each feature in the model, we have utilized SHAP algorithm for visual analysis. As illustrated in Fig. 1, feature value reflects the real value of each feature, and SHAP value reflects the contribution to the individual prognosis model, with a negative value indicating a negative contribution. Figure 1 A reveals that WBC, MPV, Mono_ratio, Baso_ratio, and Lymph_ratio are the five features that have the most significant influence on the LOF model; an increase in WBC and Mono_ratio values is associated with a poor prognosis, whereas the other three have the opposite effect. Figure 1B indicates that among the top five most important features, a rise in FIB4, GlcLR and Neu values is linked to a negative prognosis for patients, whilst the MPVLR and BLR are the opposite.

### Model comparison

The previous analysis leads us to believe that the risk value output by the DeepSurv model can be used to supplement the staging system, thereby improving the prediction efficiency. To more clearly illustrate the comparison of the prediction effects of each data combination, a DCA decision curve was utilized. The decision curve employs a horizontal axis labeled as risk threshold, with the “none” horizontal line signifying that patients are devoid of any risk. The model’s net benefit is zero in this scenario. However, if all patients are at risk, the net benefit takes the form of a negative slope backslash, as depicted by the “All” line.

**Fig. 2:**
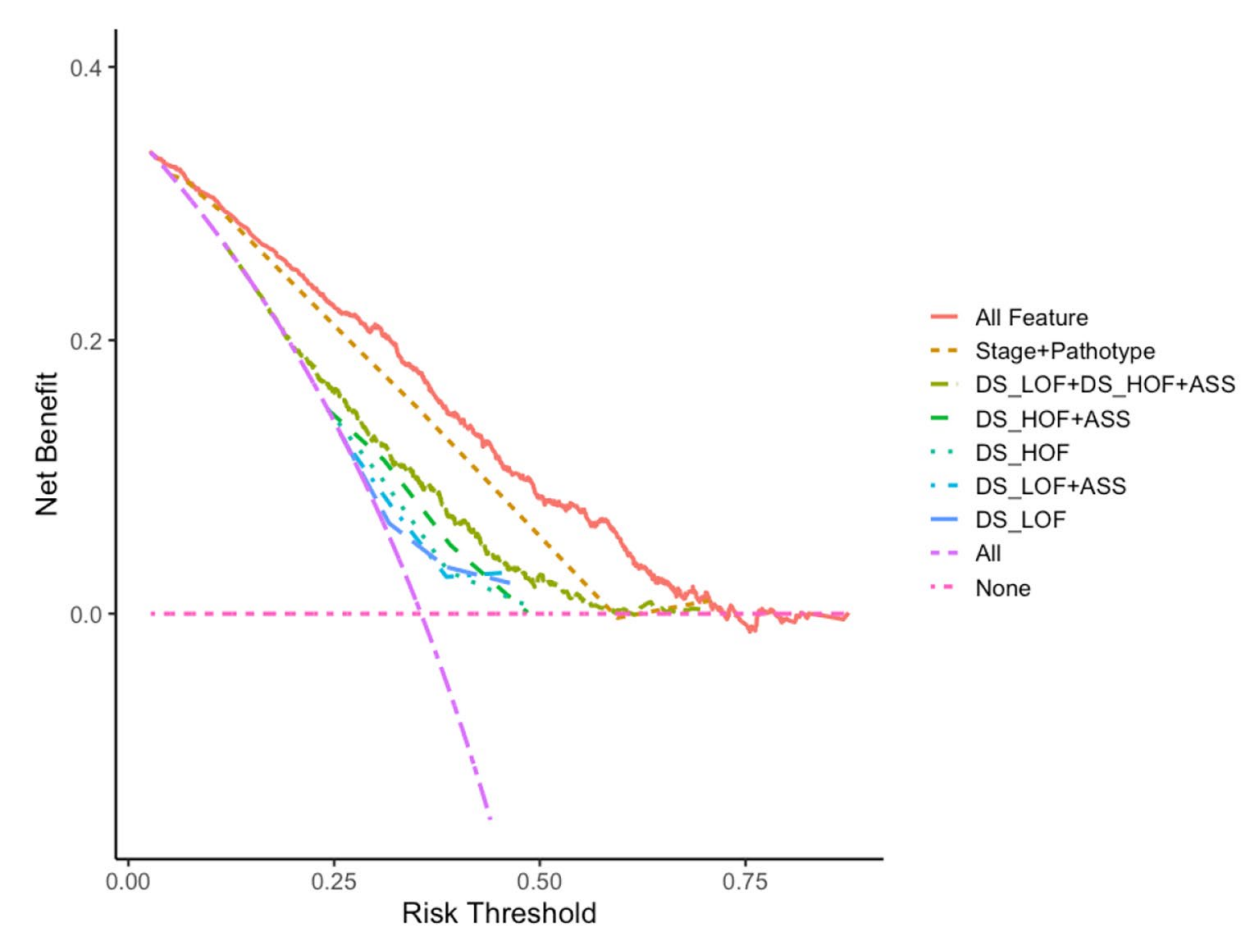
The DCA curve of different feature combinations. “DS_” means the output risk value of DeepSurv model. ASS present the combination of Age + Sex + Smoking features. All feature contains Stage + Pathotype + DS_LOF + DS_HOF + ASS features

As illustrated in Fig. 2, the risk prediction ability of HOF model is superior to that of LOF model, and the addition of ASS features can enhance the prediction efficiency of both models. However, the feature combination of DS_LOF + DS_HOF + ASS was not as effective as that of Stage + Pathotype in terms of prediction efficiency. All features (Stage + Pathotype + DS_LOF + DS_HOF + ASS)combined can provide the best prediction efficiency. It can evident that blood features can be employed as an additional factor in forecasting the risk of lung cancer patients.

### Nomogram Model

**Fig. 3:**
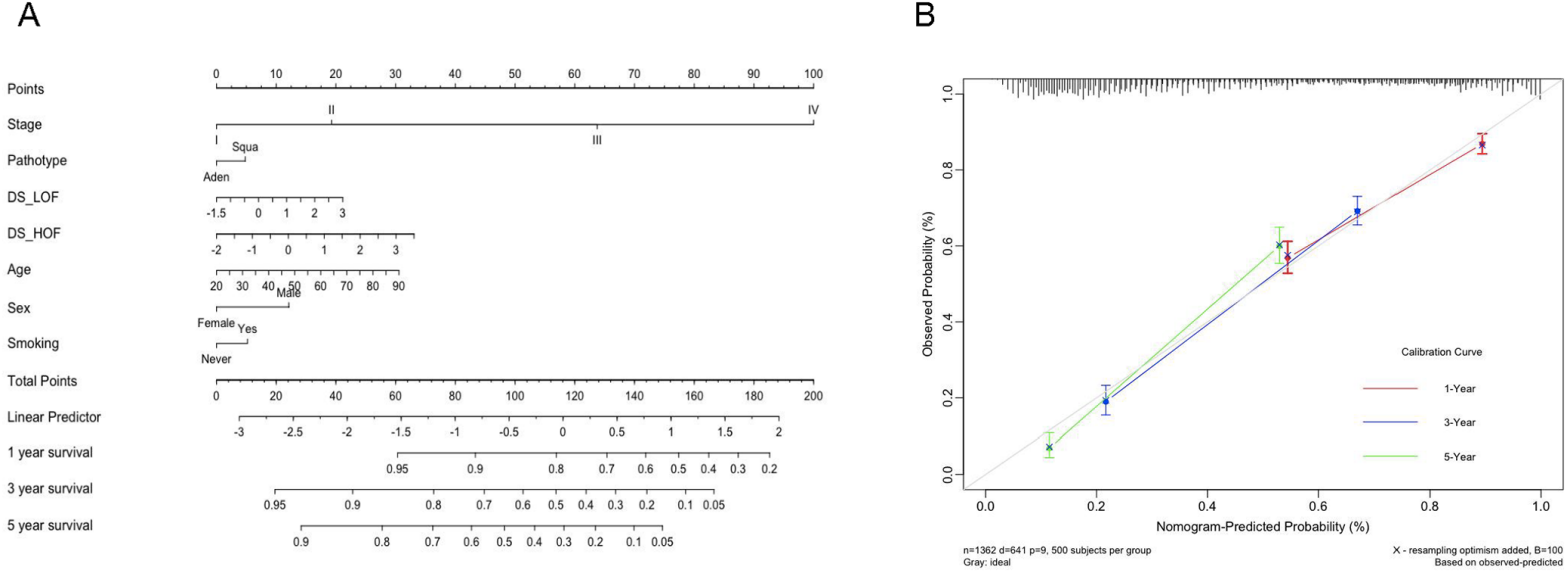
The Nomogram on OS and calibration curve of the final prognosis model. A: Nomogram for 1-, 3-, and 5-year OS. B: Calibration curve of nomogram predicting 1-, 3- and 5-year OS

Finally, a nomogram was established based on these features to obtain a more intuitive prognosis model. Figure 3 A shows that stage is still the most significant prognostic factor, followed by DS_ HOF, age, DS_ LOF, sex, pathological type and smoking status. The C-index of the model is 0.744 and the calibration curve, as seen in Fig. 3B, demonstrates its good predictive effect on lung cancer patients in 1 year and 3 years.

## Discussion

To sustain the exploration of HOFs with clinical application value and further deepen this research direction of blood test data, a sustainable expansion system needs to be established. This is the first systematic review of the blood HOF, which aims to sort and classify the existing HOFs, and to propose rules for their nomenclature.

Tables 1, 2 and 3 demonstrate that the main direction of HOF mining is to acquire features from inflammation and nutrition, such as NLR, SII, GPS, SIS and other significant HOFs which are all based on Neu, CRP, Lymph, Alb, Plt. However, for early cancer patients, their nutritional and inflammatory status may not serve as a crucial indicator. Therefore, it is suggested to start from the viewpoint of the pro- and anti-tumor balance. Tracking the changes during the treatment process could help to identify such features quickly [85]. Previous research has demonstrated that the alterations of NLR throughout treatment have a more reliable prognostic value for patients than NLR at a single point in time [79, 119].

The correlation analysis findings reveal that low-order features have little correlation with clinical features, whereas a multitude of high-order features demonstrate a correlation with clinical features. This implies that high-order features hold substantial clinical significance in cancer diagnosis and treatment. In terms of medical applications, MLR can provide insights into the likelihood of prostate cancer [16], while NLR and PLR can be utilized to predict chemotherapy response [114] and the potential for metastasis [82]. Additionally, LWR and MWR have proven to be effective in forecasting the prognosis of gastric cancer [58].

Despite numerous reports of HOFs, the clinical significance of most of them remains uncertain and the interpretability is still unsatisfactory. This study proposes that the output risk value can be utilized in addition to the staging information to optimize the prognostic efficiency, demonstrating that this usage is possible. Despite the integration of the SHAP algorithm, the inexplicable of deep learning remains unresolved. We can only ascertain the influence of the chosen features on the model’s formation, yet the weight and calculation process of each feature remain unknown.

## Conclusion

This paper’s most remarkable achievement is the sorting of reported blood HOFs, which can be used as an index for further research, and a systematic evaluation of its prediction of OS in NSCLC. However, there may still be many HOFs that have not been retrieved and included, and there is no systematic scheme for the subsequent blood HOFs mining, which will be the main goal of the research group.

## Electronic supplementary material

Below is the link to the electronic supplementary material.

Supplementary Material 1

## Data Availability

The datasets and codes used in this study are accessible from the first author or corresponding author upon reasonable request.

## Abbreviations

AJCC: The American Joint Committee on Cancer
ASS: Age + Sex + Smoking
AUC: Area under the curve
C-index: Concordance index
CT: Computed Tomography
DCA: Decision curve analysis
DS: DeepSurv
HOF: High-order feature
IQR: Interquartile range
LOF: Low-order feature
MRI: Magnetic Resonance Imaging
NSCLC: Non-small cell lung cancer
OS: Overall survival
Pathotype: Pathological Type
PET: Positron Emission Tomography
SHAP: Shapley Additive exPlanations
TNM: Tumor-lymph node-metastasis
