# Supplementary material for: The potential of high-order features of routine blood test in predicting the prognosis of non-small cell lung cancer
Source: BMC Cancer. 2023 Jun 1;23:496. doi: 10.1186/s12885-023-10990-4 (PMC10233562; doi:10.1186/s12885-023-10990-4)
Supplement: Supplementary file 1 — Supplementary Material 1 [file 12885_2023_10990_MOESM1_ESM.docx]

Supplementary Table 1. The LOFs and their normal reference intervals used in the article.

| **Abbreviation** | **LOFs** | **Refenrence Intervals** | **Units** |
| --- | --- | --- | --- |
| Alb | Albumin | 40.0-55.0 | g/L |
| ALP | Alkaline phosphatase | 40-150 | U/L |
| ALT | Alanine aminotransferase | 7.0-40.0 | U/L |
| AST | Aspartate aminotransferase | 13.0-35.0 | U/L |
| Baso | Basopil count | 0.0-0.06 | 10^9/L |
| Baso_ratio | Basopils ratio | 0-1.0 | % |
| Chol | Cholesterol | 2.33-5.20 | mmol/L |
| Crea | Creatinine | 41.0-73.0 | umol/L |
| CRP | C-reactive protein | <8 | mg/L |
| Eosin | Eosinophil count | 0.02-0.52 | 10^9/L |
| Eosin_ratio | Eosinophil ratio | 0.4-8.0 | % |
| Fib | Fibrinogen | 2.0-4.0 | g/L |
| GGT | Gamma-glutamyltransferase | 7.0-45.0 | U/L |
| Glb | Globulin | 20.0-40.0 | g/L |
| Glc | Glucose | 3.90-6.10 | mmol/L |
| HCT | Hematocrit | 33.0-45.0 | % |
| HDLC | High-density lipoprotein cholesterol | 1.1-1.74 | mmol/L |
| HGB | Hemoglobin | 115(F)/120(M)-150 | g/L |
| LDH | Lactate dehydrogenase | 109-245 | U/L |
| Lymph | Lymphocytes count | 1.1-3.2 | 10^9/L |
| Lymph_ratio | Lymphocytes ratio | 20-50 | % |
| MCH | Mean corpuscular hemoglobin | 27-34 | pg |
| MCHC | Mean corpuscular hemoglobin concentration | 316-354 | g/L |
| MCV | Mean corpuscular volume | 82-100 | fL |
| Mono | Monocyte count | 0.1-0.6 | 10^9/L |
| Mono_ratio | Monocytes ratio | 3.0-10.0 | % |
| MPV | Mean platelet volume | 9.0-17.0 | fL |
| Neu | Neutrophil count | 1.8-6.3 | 10^9/L |
| Neu_ratio | Neutrophils ratio | 40.0-75.0 | % |
| PCT | Plateletcrit; Thrombocytocrit | 0.108-0.282 | % |
| PDW | Platelets distribution width | 12.0-22.0 |  |
| Plt | Platelet count; Thrombocyte | 125-350 | 10^9/L |
| RBC | Red blood cell count; Erythrocyte count | 3.8-5.1 | 10^12/L |
| RDW_CV | RBC distribution width coefficient of variation | 11.5-14.5 | % |
| RDW_SD | RBC distribution width standard deviation | 37-54 | fL |
| TBil | Total bilirubin | 1.7-17.0 | umol/L |
| TChol | Total cholesterol | 0-5.2 | mmol/L |
| TT | Thrombin time | 12.0-16.0 | s |
| UA | Uric acid | 155.0-357.0 | umol/L |
| WBC | White blood cell count; Leukocyte count | 3.5-9.5 | 10^9/L |
| Dim | D-Dimer | <500 | ng/mL |

Supplementary Table 2. The HOFs used in the article

| **Proportional (59)** | |  | **Composite (4)** |  | **Scoring (21)** |
| --- | --- | --- | --- | --- | --- |
| AGR | MP |  | ALBI |  | ANPG |
| AISI | MPR |  | dNLR |  | HGB_X |
| AAR | MPctR |  | PALBI |  | ALB_dNLR |
| ACrR | MPVLR |  | PNI |  | SIS |
| ALRI | MWR |  |  |  | A.L.A.N |
| API | NER |  |  |  | INA |
| APR | NHL |  |  |  | CONUT |
| APRI | NLPR |  |  |  | Alb_NLR |
| BLR | NLR |  |  |  | ALBI_PLR |
| Deritis | NLLR |  |  |  | PNI_APRI |
| d_CWL | NM |  |  |  | Baso_NLR |
| ELR | NMR |  |  |  | Baso_PLR |
| ENLR | NP |  |  |  | dNLR_PNI |
| FIB4 | NPAR |  |  |  | Eosin_NLR |
| GGLR | NPR |  |  |  | Eosin_PLR |
| GlcLR | NWR |  |  |  | COP_LMR |
| GPR | PAR |  |  |  | MLR_NLR |
| HALP | PDWLR |  |  |  | coNLR_PDW |
| HII | PDWPR |  |  |  | cNPS |
| HLAN | PPR |  |  |  | COP_NLR |
| HLR | PLR |  |  |  | PLR_PNI |
| HPR | PMR |  |  |  |  |
| LA | PWR |  |  |  |  |
| LAR | SII |  |  |  |  |
| WLR | SIM |  |  |  |  |
| LMR | ULR |  |  |  |  |
| LWR | WHR |  |  |  |  |
| MAR | WMR |  |  |  |  |
| MER | WRPI |  |  |  |  |
| MGLR |  |  |  |  |  |

Supplementary Table 3. The baseline characteristics of the study cohort.

| **Characteristics** | **Overall** |
| --- | --- |
| Patient included | 1423 |
| Follow-up time (median [IQR]) | 499.00 [189.00, 1162.50] |
| status (%) |  |
| Censored | 748 (52.6) |
| Died | 675 (47.4) |
| Age (median [IQR]) | 62.00 [52.00, 67.00] |
| Sex (%) |  |
| Male | 945 (66.4) |
| Female | 478 (33.6) |
| Smoking (%) |  |
| Never | 910 (64.0) |
| Smoking | 513 (36.0) |
| Pathotype (%) |  |
| Aden | 970 (64.0) |
| Squa | 453 (36.0) |
| Stage (%) |  |
| I | 211 (14.8) |
| II | 135 (9.5) |
| III | 482 (33.9) |
| IV | 595 (41.8) |
